# Correlation between visceral fat metabolism score and erectile dysfunction: a cross-sectional study from NHANES 2001-2004

**DOI:** 10.3389/fendo.2023.1283545

**Published:** 2023-12-05

**Authors:** Lewei Huang, Heqian Liu, Lianqiang Li, Shudong Wang, Gang Sun

**Affiliations:** ^1^ General Hospital of Northern Theater Command, Shenyang, Liaoning, China; ^2^ Wuhu Hospital, East China Normal University (The Second People’s Hospital of Wuhu), Wuhu, Anhui, China

**Keywords:** visceral obesity, METS-VF, NHANES, cross-sectional studies, erectile dysfunction

## Abstract

**Backgrounds:**

The factors associated with erectile dysfunction (ED) are diverse, and obesity is a significant component. Metabolic Score for Visceral Fat (METS-VF) can assess obesity more accurately than body mass index (BMI). However, the association between METS-VF and ED remains unclear.

**Objective:**

This study aimed to investigate the association between the METS-VF and ED using National Health and Nutrition Examination Survey (NHANES) 2001-2004 data.

**Methods:**

Data were sourced from NHANES 2001-2004. The relationship between METS-VF and ED was analyzed using multivariate logistic regression, followed by subgroup analyses to identify sensitive populations. Nonlinear correlation was evaluated through smoothed curve fitting, and a threshold effect analysis validated the findings. Comparative logistic regression of the Receiver Operating Characteristic (ROC) curve assessed the diagnostic capability of METS-VF against the classical obesity index for ED.

**Results:**

The study enrolled 3625 participants, of whom 961 self-reported ED history and 360 reported severe ED. After adjusting for confounders, METS-VF exhibited a positive association with asthma prevalence (OR= 3.47, 95% CI: 2.83, 14.24). Stratification based on median METS-VF revealed higher ED prevalence in participants with elevated METS-VF (OR= 2.81,95% CI:2.32, 3.41). Nonlinear correlation was observed, with a significant association between METS-VF and ED when METS-VF exceeded 6.63. Subgroup analysis highlighted a stronger correlation in participants aged 50-85 years, Caucasians, hypertensive individuals, diabetics, and those with coronary heart disease. Sensitivity analysis using severe ED as the outcome reaffirmed the nonlinear positive association with METS-VF (OR=3.86, 95% CI:2.80,5.33), particularly when METS-VF surpassed 6.68.

**Conclusion:**

Elevated METS-VF was nonlinearly correlated with increased ED incidence. Individuals with METS-VF above 6.63 should be vigilant about heightened ED risk. Special attention should be given to participants aged 50-85 years, Caucasians, hypertensive individuals, diabetics, and those with coronary heart disease.

## 1 Introduction

Erectile dysfunction (ED) stands as a prevalent affliction among men, progressively impacting a greater number as they age (1). Before reaching the age of 40, ED prevalence ranges from 1% to 10%. Beyond 40 years, the occurrence of ED escalates dramatically to 52% (2), and an alarming 70% of men aged 70 and above contend with ED (3). Notably, Ayta IA underscored an upward trajectory in ED prevalence, projecting that over 322 million men worldwide will grapple with ED by 2025 (4). The elusive nature of ED detection, often reliant on patient self-reporting, underscores the potential for misguided medical intervention, exacerbating the condition and imposing financial strain. As ED’s prevalence surges, its socioeconomic burden deepens; current investigations estimate the expenditure on ED prevention and treatment has surpassed $15 billion (4), sans additional concealed expenses. While ED manifestations may not be life-threatening, their repercussions on relationships, mood, and overall quality of life are undeniable (5). Furthermore, despite the common perception linking ED to psychological elements like anxiety and emotional disconnect, it is imperative to acknowledge that in younger patients, erectile dysfunction can signal an underlying organic pathology (6–8).

In addition to well-defined organic pathologies such as vascular and neurological impairments, the etiology of ED is intrinsically intertwined with psychological, hormonal, and environmental factors (9). Numerous comorbidities commonly intersect with ED, encompassing diabetes mellitus, hypertension, hyperlipidemia, obesity, and testosterone deficiency (9, 10). Within the context of obesity’s broader implications, it is now apparent that obesity’s ramifications extend beyond initiating diabetes, hypertension, and hyperlipidemia, encompassing a heightened susceptibility to ED (11). Previous investigations have demonstrated that obesity can more than double the risk of ED, even when accounting for lifestyle variables (12, 13). In a multicenter inquiry, obesity, defined by waist circumference (WC) and body mass index (BMI), was shown to render individuals twice as prone to developing ED in contrast to non-obese counterparts (BMI < 30 kg/m² and WC < 102 cm). Notably, WC emerged as a superior predictor of ED compared to BMI (14). Recognizing WC’s superior sensitivity to obesity compared to BMI (15), it is prudent to establish a metric that better captures the extent of visceral fat.

Recent years have borne the concept of the metabolic score for visceral fat (METS-VF), validated in diverse systemic disorders (16, 17). METS-VF has exhibited enhanced assessment efficacy when contrasted with other established visceral fat metrics (18). Despite these strides, the relationship between METS-VF and ED risk remains inadequately elucidated. Motivated by this gap, our study aims to undertake a cross-sectional analysis, probing the interplay between METS-VF and ED prevalence using data culled from the National Health and Nutrition Examination Survey (NHANES).

## 2 Materials and methods

### 2.1 Study population

**Figure 1:**
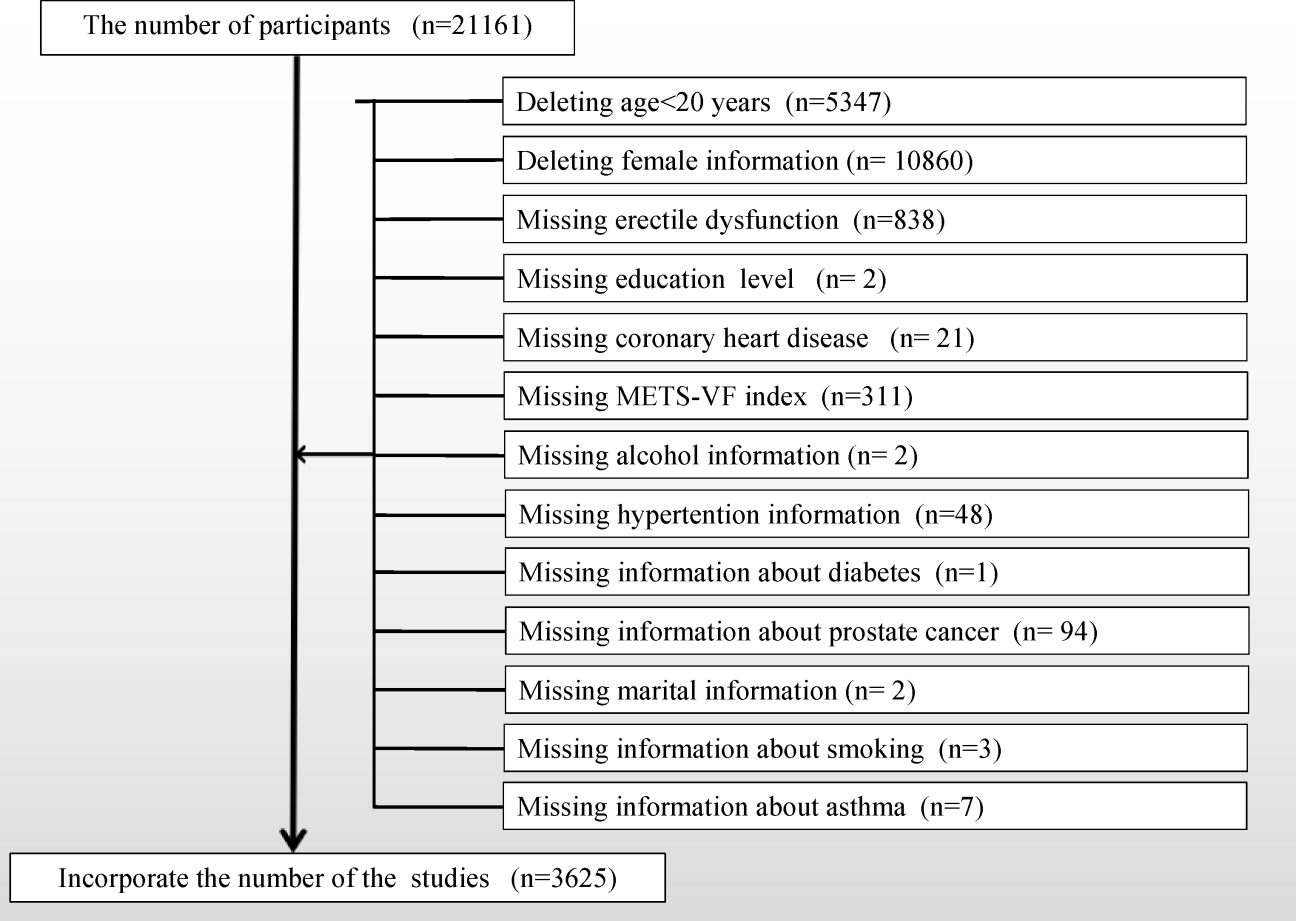
Flow chart for participants.

Utilizing the publicly available NHANES database, a comprehensive cross-sectional survey conducted biennially over the course of nearly two decades, capturing an approximate cohort size of 10,000 individuals per iteration, and overseen by the CDC, data were procured. Our study focused on the 2001 to 2004 dataset because the NHANES workgroup administered the ED questionnaire only during this time frame. Because the ED questionnaire was only administered to adult males over the age of 20, we removed participants under the age of 20 and female participants. To align with our research objectives, a stringent screening process was applied to refine the study population, as illustrated in **Figure 1**, outlining detailed inclusion and exclusion criteria. This meticulous curation culminated in the enrollment of a final cohort comprising 3625 cases. Within this cohort, 961 cases reported a history of ED, with an additional 360 cases detailing a history of severe ED.

### 2.2 Data collection and definition

The METS-VF index served as our designated exposure variable, with metabolic score for insulin resistance (METS-IR) calculated as Ln[(2×fasting glucose)+fasting triglycerides)×body mass index]/[Ln(high-density lipoprotein cholesterol)]. The formulation for METS-VF was as follows: METS-VF = 4.466 + 0.011*(Ln(METS-IR))^3 + 3.239*(Ln(WHtr))^3 + 0.319 + 0.594*(Ln(Age)). Assessment of erectile dysfunction (ED) (KIQ400) involved a structured questionnaire. Validated study-specific inquiries included, “Please describe your ability to achieve and sustain an erection adequate for sexual intercourse.” Response options encompassed “never,” “sometimes,” “usually,” and “almost always or nearly always.” We classified ED instances as respondents indicating “sometimes able” or “never able,” serving as our primary outcome measure. For sensitivity analyses, exclusive consideration was given to men who responded “never” in terms of their ability to maintain an erection (19). Prevalence of ED was treated as the outcome of interest. Literature-endorsed potential confounders, potentially impacting the association between the METS-VF index and ED, were consolidated in multivariable adjusted models. The set of covariates embraced age (years), race stratified into Mexican American, White, Black, and Other (20), educational attainment (grouped as less than high school, high school, and beyond high school levels), poverty-to-income ratio (PIR) categorized based on prior research (20), marital status (married or partnered, unmarried), alcohol consumption (derived from questionnaire ALQ101-Had at least 12 alcohol drinks/1 yr? affirmative responses identified individuals as drinkers), physical activity, cholesterol levels (mg/dl), smoking status (in accordance with questionnaire SMQ020-Smoked at least 100 cigarettes in life; affirmative responses categorized participants as smokers), hypertension, diabetes mellitus, coronary heart disease, and asthma (answered affirmatively on the questionnaire). Recognizing the potential impact of both early and advanced prostate cancer on sexual function, participants with prostate cancer were excluded. Dietary factors, comprising energy, fat, sugar, and water intake, were also considered. All participants underwent two 24-hour dietary recalls, with the average consumption from the recalls employed in our analysis. Comprehensive measurement protocols for study variables are accessible to the public at www.cdc.gov/nchs/nhanes/.

### 2.3 Handling of missing values

Continuous variables with many missing values are converted to categorical variables, and the missing variables are set as a dummy variable group and named “unclear”.

### 2.4 Statistical methods

A significance level of p<0.05 was adopted to establish statistical significance. The analyses were conducted using Empower software and R version 4.2.0. Official recommendations from the NHANES website underscored the use of appropriate sampling weights for statistical analyses. Comprehensive guidelines for weight analysis were outlined. New sampling weights were derived by dividing the 2-year weights for each survey cycle by 2 (21). The survey design R package in R was employed to process the provided dataset weights. These weights were further utilized in survey design analyses. For continuous variables, survey-weighted linear regression was employed, while categorical variables underwent survey-weighted chi-square tests. Continuous variables were presented as weighted survey means with corresponding 95% confidence intervals, and categorical variables were expressed as weighted survey proportions alongside their 95% confidence intervals. Consistent with STROBE guidelines, we established three distinct multivariate regression models. Model 1 encompassed no covariate adjustments. Model 2 integrated adjustments for race, education level, and marital status. Model 3 encompassed adjustments for all variables excluding age (as its effect was concurrently captured along with the unadjusted METS-VF index in Model 3). To assess robustness, sensitivity analyses converted the METS-VF from a continuous variable to a bicategorical variable. A linear trend test was applied using the two quartiles of METS-VF as a continuous entity. Further validation was pursued through inverse probability weighting. Addressing the potential nonlinearity of METS-VF in relation to ED, we employed a generalized additive model (GAM) and smooth curve fitting. If nonlinearity patterns emerged, a two-segment linear regression model (segmented regression model) was engaged, fitting each interval and quantifying threshold effects. Finally, the predictive efficacy of WWI, BMI, and WC concerning ED was evaluated via receiver operating characteristic (ROC) curves and area under the curve (AUC) calculations (22).

## 3 Results

### 3.1 Baseline characteristics of the participants

**Table 1** presents the demographic baseline features of the enrolled participants. The study encompassed 3625 participants, whose weighted attributes were delineated based on ED presence. Notably, substantial distinctions were observed in baseline characteristics, with the exception of race and estrogen levels. Specifically, those afflicted with ED exhibited a propensity toward higher age, blood cholesterol levels, BMI, waist circumference, and METS-VF values, in addition to a higher incidence of hypertension, asthma, diabetes, and coronary heart disease.

**Table 1:** Baselines characteristics of participants, weighted.

Characteristic	Non-ed formers (n=2664)	Ed formers (n=961)	P-value
Age (years)	41.21 (40.66,41.77)	60.27 (59.29,61.26)	<0.0001
BMI (kg/m^2^)	27.91 (27.66,28.15)	29.01 (28.45,29.57)	0.0024
Serum Cholesterol (mg/dl)	201.76 (199.80,203.71)	200.29 (195.54,205.04)	0.562
Waist (cm)	99.10 (98.49,99.71)	105.28 (103.85,106.70)	<0.0001
METS-VF	6.17 (6.15,6.19)	6.65 (6.61,6.68)	<0.0001
Race (%)			0.2586
Mexican American	7.61 (5.81,9.92)	7.01 (4.32,11.18)	
White	78.71 (75.22,81.83)	81.51 (76.51,85.65)	
Black	9.59 (7.62,12.01)	8.41 (6.26,11.22)	
Other Race	4.09 (3.14,5.30)	3.08 (1.95,4.82)	
Education Level (%)			<0.0001
Less than high school	13.33 (12.02,14.76)	28.45 (23.95,33.42)	
High school	27.78 (25.59,30.08)	23.80 (20.55,27.38)	
More than high school	58.89 (56.41,61.34)	47.75 (43.41,52.13)	
Marital Status (%)			<0.0001
Cohabitation	68.59 (65.78,71.27)	77.06 (73.88,79.96)	
Solitude	31.41 (28.73,34.22)	22.94 (20.04,26.12)	
Alcohol (%)			0.0593
Yes	84.41 (80.02,87.98)	81.10 (76.73,84.81)	
No	15.59 (12.02,19.98)	18.90 (15.19,23.27)	
High Blood Pressure (%)			<0.0001
Yes	21.16 (18.85,23.68)	50.17 (46.95,53.40)	
No	78.84 (76.32,81.15)	49.83 (46.60,53.05)	
Diabetes (%)			<0.0001
Yes	3.80 (2.95,4.89)	22.04 (18.25,26.37)	
No	96.20 (95.11,97.05)	77.96 (73.63,81.75)	
Smoked (%)			<0.0001
Yes	54.25 (51.27,57.19)	70.58 (67.08,73.86)	
No	45.75 (42.81,48.73)	29.42 (26.14,32.92)	
Asthma (%)			0.0023
Yes	10.96 (9.67,12.40)	7.34 (5.68,9.44)	
No	89.04 (87.60,90.33)	92.66 (90.56,94.32)	
Coronary Artery Disease (%)			<0.0001
Yes	2.54 (1.93,3.33)	14.83 (11.90,18.34)	
No	97.46 (96.67,98.07)	85.17 (81.66,88.10)	
PIR (%)			<0.0001
< 1.3	14.73 (12.84,16.85)	16.96 (13.92,20.50)	
≥ 1.3 < 3.5	32.04 (29.61,34.57)	40.10 (36.03,44.31)	
≥ 3.5	48.29 (45.07,51.53)	38.12 (33.58,42.87)	
Unclear	4.94 (3.72,6.52)	4.82 (3.26,7.08)	
Total Kcal (%)			<0.0001
Lower	36.90 (34.25,39.63)	58.14 (53.05,63.05)	
Higher	58.15 (55.31,60.94)	37.58 (32.11,43.39)	
Unclear	4.95 (3.98,6.14)	4.28 (2.78,6.54)	
Total Sugar (%)			<0.0001
Lower	39.32 (37.05,41.64)	55.07 (51.45,58.63)	
Higher	51.30 (48.58,54.00)	34.79 (30.22,39.66)	
Unclear	9.38 (7.93,11.07)	10.14 (7.78,13.11)	
Total Water (%)			0.004
Lower	47.31 (43.58,51.07)	54.63 (47.73,61.35)	
Higher	47.74 (43.93,51.57)	41.09 (34.74,47.75)	
Unclear	4.95 (3.98,6.14)	4.28 (2.78,6.54)	
Total Fat (%)			<0.0001
Lower	38.15 (35.17,41.23)	52.49 (48.06,56.88)	
Higher	56.90 (53.59,60.14)	43.23 (38.31,48.29)	
Unclear	4.95 (3.98,6.14)	4.28 (2.78,6.54)	
Testosterone (%)			0.0444
Lower	7.70 (6.63,8.93)	10.84 (8.22,14.16)	
Higher	8.40 (6.87,10.24)	6.54 (4.76,8.93)	
Unclear	83.90 (81.84,85.76)	82.62 (79.06,85.68)	
Estradiol (%)			0.4484
Lower	7.60 (6.53,8.83)	9.47 (7.25,12.27)	
Higher	8.50 (7.01,10.28)	7.91 (5.44,11.36)	
Unclear	83.90 (81.84,85.76)	82.62 (79.06,85.6	

For continuous variables: survey-weighted mean (95% CI), P-value was by survey-weighted linear regression (svyglm).

For categorical variables: survey-weighted percentage (95% CI), P-value was by survey-weighted Chi-square test (svytable).

### 3.2 Higher METS-VF indices were associated with higher prevalence of ED

Diverse regression analyses, encompassing distinct adjustments to account for confounding influences on the correlation, illuminated a consistent positive linkage between the METS-VF index and ED across both raw and meticulously adjusted models. Within the fully adjusted model, each incremental unit elevation in the METS-VF index manifested as a substantial 247% surge in ED risk (OR=3.47, 95% CI: 2.83, 14.24). Upon categorizing the METS-VF index into two quartiles, logistic regression highlighted a notable 1.81-fold escalation in ED risk prevalence within the highest group, relative to the lowest METS-VF index category (OR=2.81, 95% CI: 2.32, 3.41). To further substantiate these findings, an inverse probability weighted analysis was performed, post-METS-VF dichotomization. **Supplementary Table 1** demonstrates the equalization of baseline attributes between the two groups, following which inverse probability weighted logistic regression unveiled a statistically significant 95% upsurge in ED risk prevalence within the highest METS-VF index stratum, contrasted with the lowest group (OR=1.95, 95% CI: 1.50, 2.54) (**Table 2**, **Supplementary Table 1**).

**Table 2:** Logistic regression analysis between METS-VF index with ED prevalence.

Characteristic	Model 1 OR (95% CI)	Model 2 OR (95% CI)	Model 3 OR (95% CI)	Model 4 OR (95% CI)
METS-VF	6.10 (5.06, 7.37)	5.94 (4.90, 7.20)	3.47 (2.83, 4.24)	3.21 (2.19, 4.70)^★^
Categories				
Lower (3.40-6.47)	1	1	1	1
Higher (6.47-7.38)	4.65 (3.93, 5.49)	4.53 (3.80, 5.39)	2.81 (2.32, 3.41)	1.95 (1.50, 2.54)^★^

Model 1 was adjusted for no covariates;

Model 2 was adjusted for race, marital status and education;

Model 3 was adjusted for covariates in Model 2+diabetes,blood pressure, PIR, total water,total kcal,total sugar, total fat, smoked, physical activity, alcohol use, serum cholesterol, coronary artery disease, asthma, testosterone and estradiol were adjusted.

Model 4:The covariates that need to be adjusted were consistent with those in Model 3.

^★^ = IPTW analysis only in model 4.

**Figure 2:**
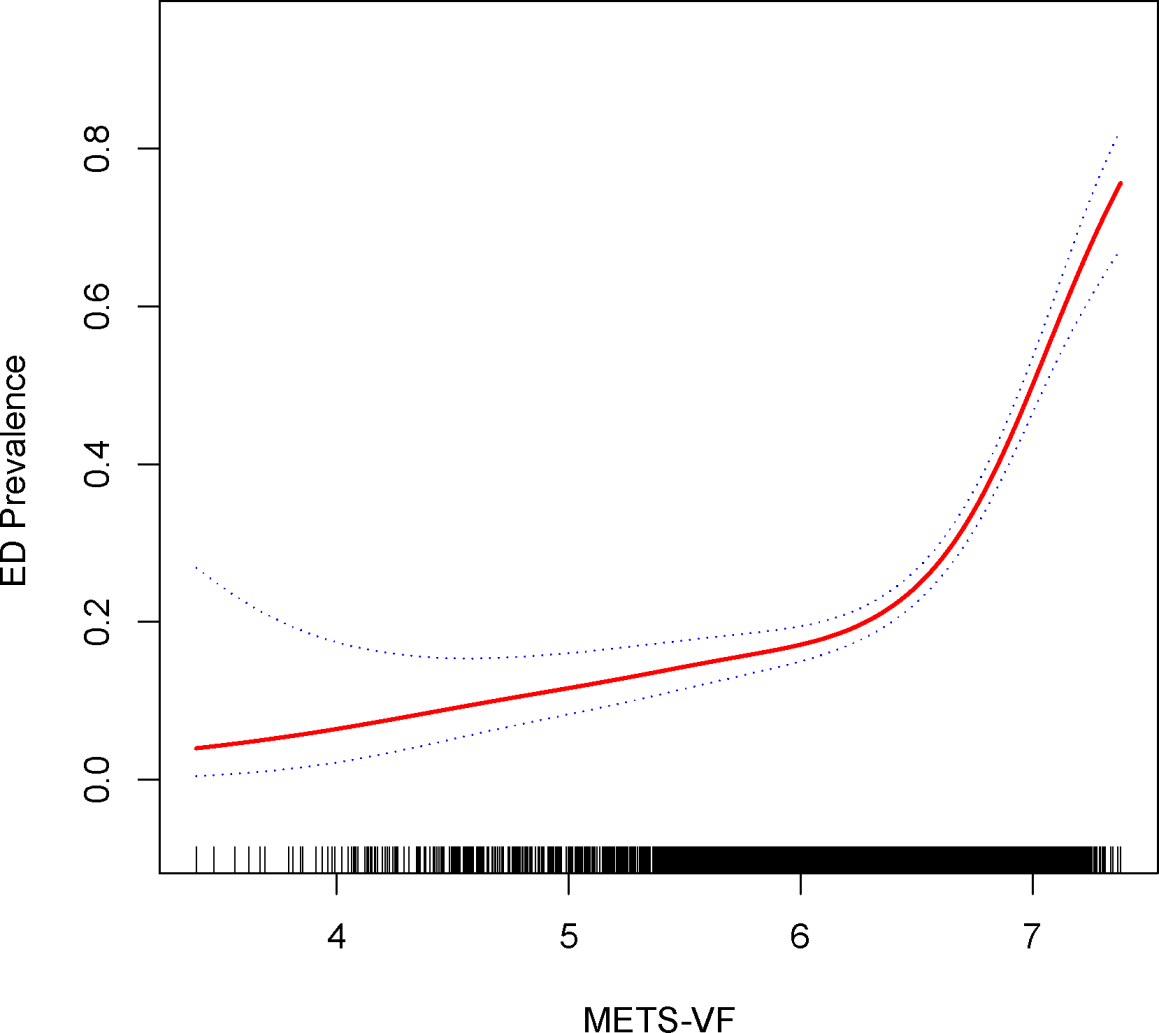
Density dose-response relationship between METS-VF index with ED prevalence. The area between the upper and lower dashed lines is represented as 95% CI. Each point shows the magnitude of the index and is connected to form a continuous line. Adjusted for all covariates except effect modifier.

Exploration of the METS-VF index’s relationship with ED was extended via generalized additive modeling and smoothed curve fitting. Our findings underscored a nonlinear positive correlation between the METS-VF index and ED (**Figure 2**). Subsequent application of a likelihood ratio test revealed a discernible threshold effect of METS-VF on ED, with the risk of ED onset exhibiting a sharp increase post-METS-VF index surpassing 6.63 (**Table 3**).

**Table 3:** Two-piecewise linear regression and logarithmic likelihood ratio test explained the threshold effect analysis of METS-VF index with ED prevalence.

METS-VF Index	ULR Test	PLR Test	LRT test
	OR (95% CI)	OR (95% CI)	P value
< 6.63	3.47 (2.83, 4.24)	1.82 (1.42, 2.33)	< 0.0001
≥ 6.63		20.28 (11.63, 35.38)	

ULR, univariate linear regression; PLR, piecewise linear regression; LRT, logarithmic likelihood ratio test, statistically significant: p<0.05.

### 3.3 Subgroup analysis

Subgroup analyses were performed to assess the robustness of the association between the METS-VF index and ED. Results Age <50 years group (OR=1.33, 95% CI:0.95, 1.88), age 50-85 years group (OR=2.05, 95% CI:1.54, 2.73), Mexican American group (OR=2.71, 95% CI:1.70, 4.33), White group (OR=5.01, 95% CI:3.76, 6.67), black group (OR=2.19, 95% CI:1.46, 3.29), others group (OR=1.76, 95% CI:0.53, 5.84), hypertensive group (OR=3.64, 95% CI:2.56, 5.18),non-hypertensive group (OR=3.38, 95% CI:2.63, 4.25), diabetes group (OR=3.79, 95% CI:1.86,7.71),Non-diabetic group (OR=3.49, 95% CI:2.82, 4.32),Coronary heart disease group (OR=3.82, 95% CI:1.41,10.36),Non-coronary heart disease group (OR=3.47, 95% CI:2.82,4.27) **Table 4**.

**Table 4:** Subgroup regression analysis between METS-VF index with ED prevalence.

Characteristic	Model 1 OR (95% CI)	Model 2 OR (95% CI)	Model 3 OR (95% CI)
Stratified by age (years)			
20-49	1.44 (1.07, 1.94)	1.47 (1.07, 2.02)	1.33 (0.95, 1.88)
50-85	2.74 (2.10, 3.57)	2.76 (2.11, 3.62)	2.05 (1.54, 2.73)
Stratified by race			
Mexican American	4.94 (3.25, 7.49)	4.90 (3.21, 7.49)	2.71 (1.70, 4.33)
White	8.88 (6.77, 11.66)	8.53 (6.49, 11.21)	5.01 (3.76, 6.67)
Black	3.55 (2.49, 5.06)	3.60 (2.48, 5.21)	2.19 (1.46, 3.29)
Other Race	2.24 (0.90, 5.55)	1.94 (0.75, 5.04)	1.76 (0.53, 5.84)
Stratified by hypertension			
Yes	4.78 (3.44, 6.65)	4.65 (3.32, 6.49)	3.64 (2.56, 5.18)
No	4.58 (3.62, 5.80)	4.40 (3.45, 5.62)	3.38 (2.63, 4.35)
Stratified by diabetes			
Yes	4.09 (2.26, 7.40)	3.77 (2.06, 6.90)	3.79 (1.86, 7.71)
No	5.21 (4.26, 6.37)	5.09 (4.14, 6.25)	3.49 (2.82, 4.32)
Stratified by CVD			
Yes	3.43 (1.55, 7.55)	3.44 (1.50, 7.87)	3.82 (1.41, 10.36)
No	5.64 (4.64, 6.86)	5.58 (4.57, 6.82)	3.47 (2.82, 4.27)

Model 1 was adjusted for no covariates;

Model 2 was adjusted for race,marital status and education;

Model 3 adjusted for all covariates except effect modifier.

### 3.4 Sensitivity analysis

**Figure 3:**
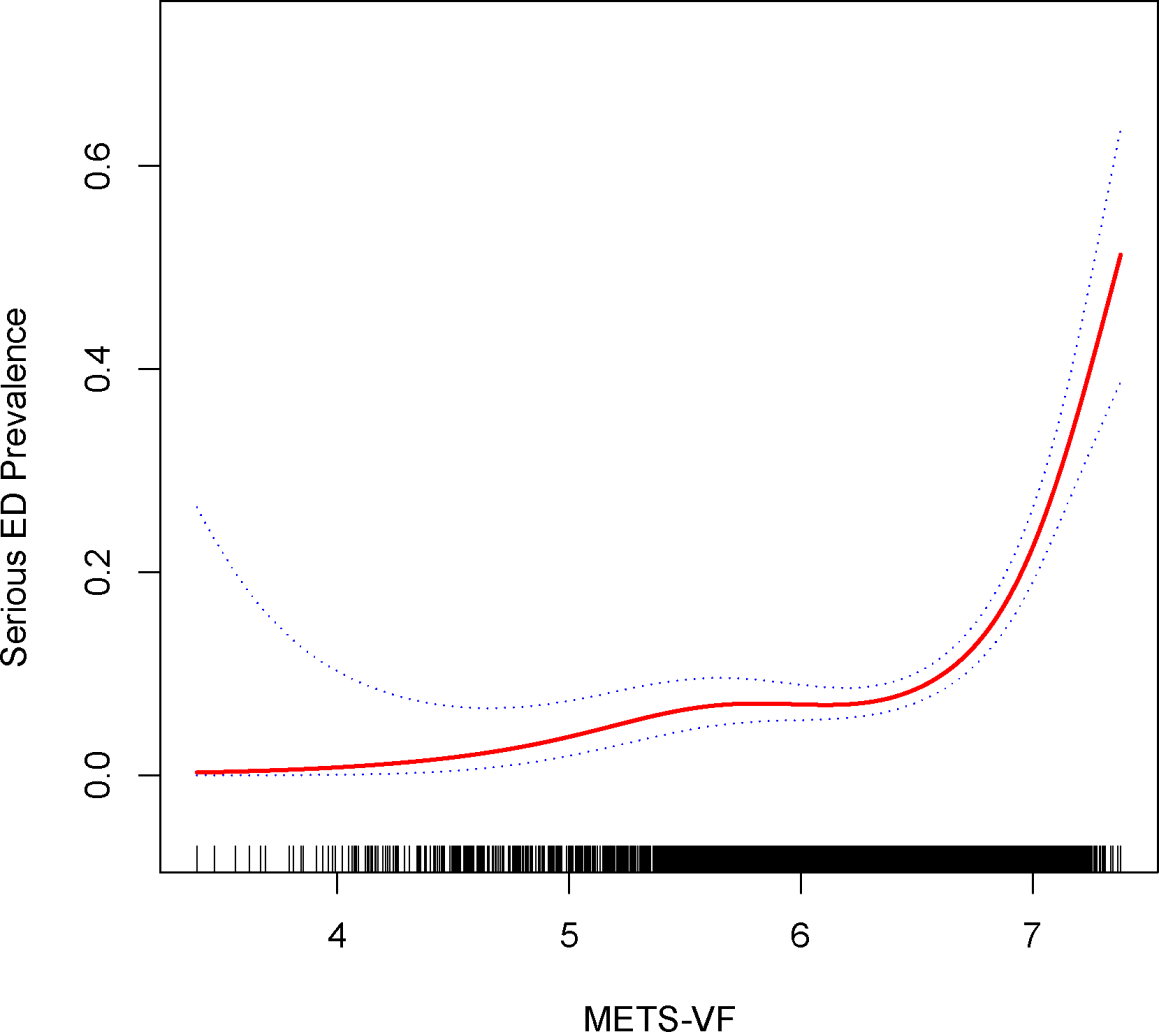
Density dose-response relationship between METS-VF index with serious ED prevalence. The area between the upper and lower dashed lines is represented as 95% CI. Each point shows the magnitude of the index and is connected to form a continuous line. Adjusted for all covariates except effect modifier.

For sensitivity analysis, we categorized participants responding as ‘never able’ to maintain an erection as individuals with more pronounced ED severity. As demonstrated in **Table 5**, affirmative associations were evident across all models. In Model 3, each additional unit increment in METS-VF exhibited a substantial 286% surge in the risk of ED (OR=3.86, 95% CI: 2.80, 5.33). Findings stemming from the inverse probability weighting technique depicted a 0.65-fold augmentation in the risk of ED prevalence for every elevated METS-VF unit (OR=1.65, 95% CI: 1.10, 2.48). Employing smoothed curve fitting and a generalized additive model, we unraveled a nonlinear positive correlation between METS-VF and more severe ED (**Figure 3**), with the most favorable inflection point detected at 6.68 (**Table 6**).

**Table 5:** Logistic regression analysis between METS-VF index with serious ED prevalence.

Characteristic	Model 1 OR (95% CI)	Model 2 OR (95% CI)	Model 3 OR (95% CI)	Model 4 OR (95% CI)
METS-VF	7.98 (5.86, 10.87)	6.97 (5.11, 9.49)	3.86 (2.80, 5.33)	2.26 (1.12, 4.53)^★^
Categories				
Lower (3.40-6.47)	1	1	1	1
Higher (6.47-7.38)	4.83 (3.68, 6.34)	4.35 (3.29, 5.74)	2.66 (1.96, 3.59)	1.65 (1.10, 2.48)^★^

Model 1 was adjusted for no covariates;

Model 2 was adjusted for race,marital status and education;

Model 3 was adjusted for covariates in Model 2+diabetes,blood pressure, PIR, total water,total kcal, total sugar, total fat, smoked, physical activity, alcohol use, serum cholesterol, coronary artery disease, asthma, testosterone and estradiol were adjusted.

Model 4:The covariates that need to be adjusted were consistent with those in Model 3.

^★^ = IPTW analysis only in model 4.

**Table 6:** Two-piecewise linear regression and logarithmic likelihood ratio test explained the threshold effect analysis of METS-VF index with serious ED prevalence.

METS-VF Index	ULR Test	PLR Test	LRT test
	OR (95% CI)	OR (95% CI)	P value
< 6.68	3.86 (2.80, 5.33)	1.63 (1.10, 2.41)	<0.0001
≥ 6.68		23.75 (11.31, 49.85)	

### 3.5 METS-VF was a stronger predictor of ED than BMI and WC

**Figure 4:**
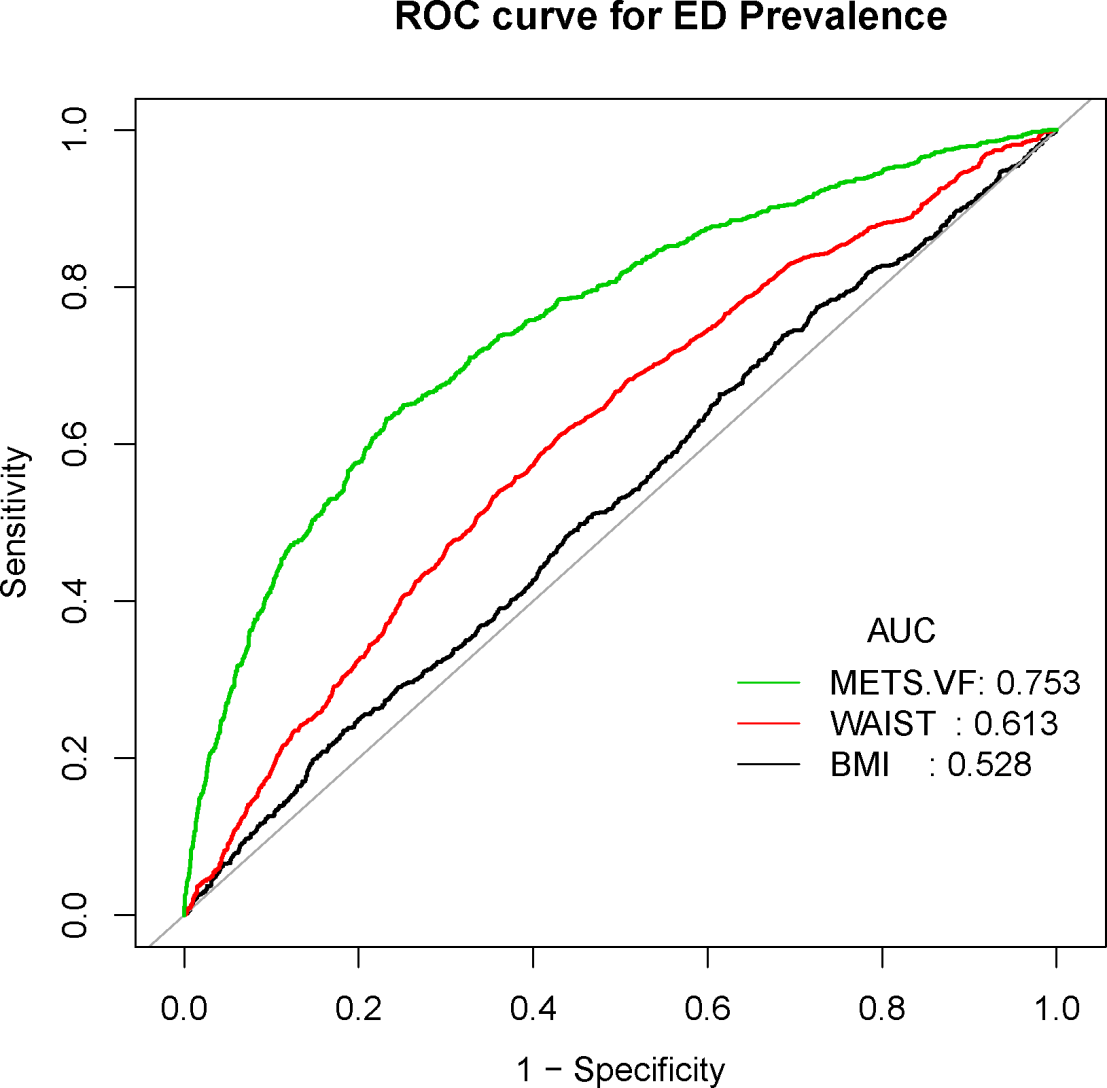
Receiver operating characteristic (ROC) curve analysis for predicting ED. Comparison of area under curve (AUC) value between WWI and BMI, WC.

**Figure 5:**
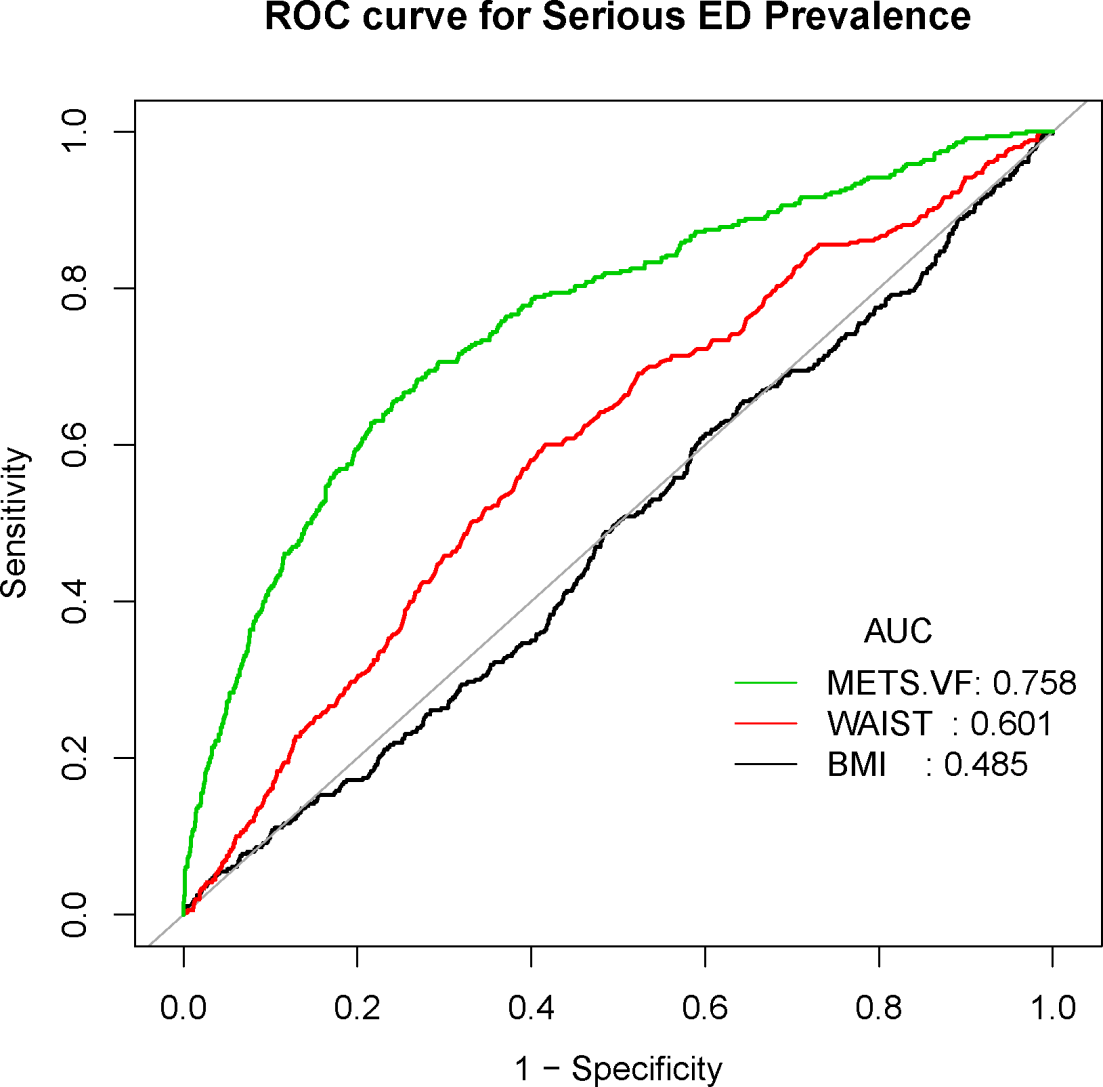
Receiver operating characteristic (ROC) curve analysis for predicting serious ED. Comparison of area under curve (AUC) value between WWI and BMI, WC.

Conclusively, we delved into the diagnostic potential of METS-VF, BMI, and WC concerning ED. Our analysis unveiled noteworthy findings – the AUC values for METS-VF distinctly surpassed those of BMI and WC, applicable to both ED and more severe ED cases (**Figures 4**, **5**).

## 4 Discussion

To our knowledge, this marks the inaugural cross-sectional investigation to gauge the connection between METS-VF and the prevalence of ED utilizing a representative cohort of US adults. Our findings underscore a nonlinear positive linkage between METS-VF and ED prevalence, extending this correlation to participants with more pronounced ED severity. Furthermore, we exhibit the heightened predictive utility of METS-VF relative to conventional obesity benchmarks such as BMI and WC.

Earlier research has ascertained that age, smoking, sedentary behavior, and obesity exhibit robust correlations with ED development, with a subset of cases (20%) attributed to psychological factors (23). The global economic shift has facilitated the widespread emergence of obesity as a significant public health concern due to a Westernized dietary pattern adopted by populations worldwide (24). Despite this, the precise mechanistic underpinnings of obesity-related ED remain elusive (11), motivating researchers to focus on effective preventive strategies for ED within the obese demographic. Consequently, accurate assessment of an individual’s genuine obesity status has become paramount. In this context, researchers have increasingly cast doubt on the adequacy of BMI as a precise marker, instead viewing it as a rudimentary indicator for identifying obesity or overweight status (25). This arises from BMI’s inability to differentiate between fat mass and lean (muscle) mass, while also failing to elucidate localized fat distribution patterns (26). While WC exhibits better sensitivity to obesity, particularly abdominal obesity (15), its use as a solitary measure remains inadequate for distinguishing between subcutaneous and visceral fat deposits (27, 28).

Prior research has indicated that variables such as age, sex, waist-to-height ratio (WhtR), METS-IR, and fasting glucose (FPG) and triglycerides (TG) within the METS-VF hold promise as indicators of intra-abdominal fat content response (18). The accumulation of intra-abdominal fat is notably associated with more perilous health implications than fat accumulation in other regions. In our study, we confirm a positive correlation between METS-VF and ED prevalence, suggesting potential advantages for METS-VF in assessing ED prevalence. First, it is widely established that the prevalence of ED escalates with advancing age, notably being considerably higher in men above 40 years of age compared to their younger counterparts (4). Our investigation similarly highlights the elevated ED risk in men over 40 years. Secondly, earlier cohort-based studies have demonstrated diabetes to be the leading risk factor for ED, associated with a 1.3- to 3-fold amplified risk of ED onset, even after accounting for diabetes type and age (29–31). Although METS-VF isn’t a diagnostic tool for diabetes or its type, the inclusion of fasting glucose contributes significantly to diabetes presence assessment. Notably, the METS-IR effectively reflects insulin resistance degree and possesses advantages in assessing adverse outcomes in type 2 diabetes (32). This aligns with prior observations that insulin resistance status might contribute to ED development through impaired vascular nitric oxide (NO) production and vasodilation, underscoring the need to incorporate insulin resistance diagnosis and management into ED care preceding diabetes onset (33). Third, though direct comparisons of WhtR and WC in predicting ED remain lacking, WhtR has demonstrated superior predictive power for diabetes mellitus, hypertension, and cardiovascular disease relative to WC (34). Furthermore, our study substantiates that METS-VF outperforms WC in diagnosing ED, accentuating the robustness of our findings.

Lastly, the generation of active adipokines stemming from abnormal visceral adiposity emerges as a pivotal driver of chronic inflammation within the body (35). A potent connection exists between inflammation and ED development, particularly pronounced in obese individuals (36). Nonetheless, preceding investigations have underscored that abnormal lipid profiles wield a stronger correlation with ED severity compared to inflammatory markers (37). Moreover, while the precise mechanisms linking obesity and ED development remain elusive, it is well-accepted that obesity can trigger diminished androgen production (e.g., total testosterone), heightened conversion of androgens to estrogens, and hypogonadotropic hypogonadism (11, 38, 39). These adverse effects associated with obesity are similarly evident in individuals with diabetes mellitus, insulin resistance, and dyslipidemia (40, 41).

The present study holds several strengths that contribute to its significance. Foremost, it stands as the inaugural cross-sectional examination delving into the interplay between visceral fat distribution and the prevalence of ED through the application of a simplified scoring system. Moreover, our study is underscored by the selection of a robust and representative sample, further bolstering its merit. Nevertheless, the study also carries certain limitations that warrant acknowledgment. First and foremost, the inherent nature of cross-sectional studies restricts our ability to deduce causality. Establishing whether a causal link exists between METS-VF and ED, and deciphering the unidirectional or bidirectional nature of this association, demands further substantiation in subsequent investigations. Second, the assessment of ED in this study was reliant upon self-reported participant surveys, inherently susceptible to recall bias. Consequently, prospective follow-up studies are imperative to provide more robust insights. Third, the potential influences stemming from ED and METS-VF are multifaceted. While extensive endeavors were undertaken to encompass relevant covariates within our model for adjustments, it remains an ongoing challenge to entirely mitigate the potential impact of other covariates that may be at play.

## 5 Conclusions

Our study harnessed data derived from a representative U.S. population sample, effectively unveiling a robust and affirmative linkage between METS-VF and the prevalence of ED. Notably, our findings indicate that METS-VF levels surpassing 6.63 and 6.68 correspondingly usher in a notable surge in the risk of ED and heightened ED severity. Additionally, this observed positive correlation highlights the need for heightened vigilance among participants aged 50-85 years, those of Caucasian ethnicity, individuals with hypertension, diabetes, and coronary heart disease.

## Data availability statement

The raw data supporting the conclusions of this article will be made available by the authors, without undue reservation.

## Ethics statement

The NCHS Research Ethics Review Committee approved the NHANES survey protocol, and all participants of the study provided informed written consent. The NHANES database is open to the public and therefore the ethical review of this study was exempt.

## Author contributions

LH: Conceptualization, Investigation, Writing – original draft. HL: Data curation, Investigation, Methodology, Writing – original draft. LL: Formal analysis, Project administration, Writing – review & editing. SW: Data curation, Software, Validation, Writing – original draft. GS: Conceptualization, Investigation, Methodology, Software, Supervision, Validation, Writing – original draft, Writing – review & editing.
