# Supplementary material for: Correlation between visceral fat metabolism score and erectile dysfunction: a cross-sectional study from NHANES 2001-2004
Source: Front Endocrinol (Lausanne). 2023 Dec 5;14:1283545. doi: 10.3389/fendo.2023.1283545 (PMC10732023; doi:10.3389/fendo.2023.1283545)
Supplement: Supplementary file 1 [file Table_1.doc]

Supplementary Table 1.Comparison of baseline data for METS-VF classification by using IPTW.

| Characteristic | Lower | Higher | P value |
| --- | --- | --- | --- |
| Serum Cholesterol(mg/dl) | 201.99 ± 43.30 | 201.55 ± 42.81 | 0.7572 |
| Race (%) |  |  | 0.855 |
| Mexican American | 0.206 | 0.199 |  |
| White | 0.567 | 0.575 |  |
| Black | 0.195 | 0.191 |  |
| Other Race | 0.031 | 0.035 |  |
| Education Level (%) |  |  | 0.4702 |
| Less than high school | 0.278 | 0.264 |  |
| High school | 0.238 | 0.252 |  |
| More than high school | 0.484 | 0.484 |  |
| Marital Status (%) |  |  | 0.3477 |
| Cohabitation | 0.71 | 0.696 |  |
| Solitude | 0.29 | 0.304 |  |
| Asthma (%) |  |  | 0.7275 |
| Yes | 0.91 | 0.906 |  |
| No | 0.09 | 0.094 |  |
| Alcohol (%) |  |  | 0.5043 |
| Yes | 0.831 | 0.839 |  |
| No | 0.169 | 0.161 |  |
| High Blood Pressure (%) |  |  | 0.0539 |
| Yes | 0.346 | 0.313 |  |
| No | 0.654 | 0.687 |  |
| Diabetes(%) |  |  | 0.0612 |
| Yes | 0.148 | 0.105 |  |
| No | 0.852 | 0.895 |  |
| Smoked(%) |  |  | 0.128 |
| Yes | 0.615 | 0.591 |  |
| No | 0.385 | 0.409 |  |
| Coronary Artery Disease (%) |  |  | 0.0584 |
| Yes | 0.1 | 0.064 |  |
| No | 0.9 | 0.936 |  |
| PIR(%) |  |  | 0.7337 |
| < 1.3 | 0.214 | 0.226 |  |
| ≥ 1.3 < 3.5 | 0.383 | 0.369 |  |
| ≥ 3.5 | 0.35 | 0.355 |  |
| Unclear | 0.053 | 0.05 |  |
| Total Kcal (%) |  |  | 0.142 |
| Lower | 0.508 | 0.476 |  |
| Higher | 0.441 | 0.473 |  |
| Unclear | 0.051 | 0.05 |  |
| Total Sugar (%) |  |  | 0.6455 |
| Lower | 0.473 | 0.458 |  |
| Higher | 0.436 | 0.45 |  |
| Unclear | 0.091 | 0.092 |  |
| Total Water (%) |  |  | 0.7645 |
| Lower | 0.497 | 0.485 |  |
| Higher | 0.453 | 0.465 |  |
| Unclear | 0.051 | 0.05 |  |
| Total Fat (%) |  |  | 0.827 |
| Lower | 0.485 | 0.475 |  |
| Higher | 0.464 | 0.474 |  |
| Unclear | 0.051 | 0.05 |  |
| Testosterone (%) |  |  | 0.4616 |
| Lower | 0.07 | 0.081 |  |
| Higher | 0.079 | 0.079 |  |
| Unclear | 0.851 | 0.84 |  |
| Estradiol (%) |  |  | 0.5924 |
| Lower | 0.077 | 0.081 |  |
| Higher | 0.072 | 0.08 |  |
| Unclear | 0.851 | 0.84 |  |

Mean +/- SD for: Continuous variables.P value was calculated by weighted linear regression model.

% for: Categorical variables. P value was calculated by weighted chi-square test.
